# Overproducing the BAM complex improves secretion of difficult-to-secrete recombinant autotransporter chimeras

**DOI:** 10.1186/s12934-021-01668-2

**Published:** 2021-09-06

**Authors:** Trang H. Phan, Coen Kuijl, Dung T. Huynh, Wouter S. P. Jong, Joen Luirink, Peter van Ulsen

**Affiliations:** 1 Department of Molecular Microbiology, Amsterdam Institute of Molecular and Life Sciences, Vrije Universiteit Amsterdam, Amsterdam, The Netherlands; 2 Medical Microbiology and Infection Control, Amsterdam Institute of Infection & Immunity, Amsterdam UMC, Amsterdam, The Netherlands; 3 Abera Bioscience AB, Solna, Sweden

**Keywords:** Type V secretion systems, Hbp, Surface display, BAM complex, Outer membrane proteins

## Abstract

Monomeric autotransporters have been used extensively to transport recombinant proteins or protein domains to the cell surface of Gram-negative bacteria amongst others for antigen display. Genetic fusion of such antigens into autotransporters has yielded chimeras that can be used for vaccination purposes. However, not every fusion construct is transported efficiently across the cell envelope. Problems occur in particular when the fused antigen attains a relatively complex structure in the periplasm, prior to its translocation across the outer membrane. The latter step requires the interaction with periplasmic chaperones and the BAM (β-barrel assembly machinery) complex in the outer membrane. This complex catalyzes insertion and folding of β-barrel outer membrane proteins, including the β-barrel domain of autotransporters. Here, we investigated whether the availability of periplasmic chaperones or the BAM complex is a limiting factor for the surface localization of difficult-to-secrete chimeric autotransporter constructs. Indeed, we found that overproduction of in particular the BAM complex, increases surface display of difficult-to-secrete chimeras. Importantly, this beneficial effect appeared to be generic not only for a number of monomeric autotransporter fusions but also for fusions to trimeric autotransporters. Therefore, overproduction of BAM might be an attractive strategy to improve the production of recombinant autotransporter constructs.

**Supplementary Information:**

The online version contains supplementary material available at 10.1186/s12934-021-01668-2.

## Introduction

**Fig. 1:**
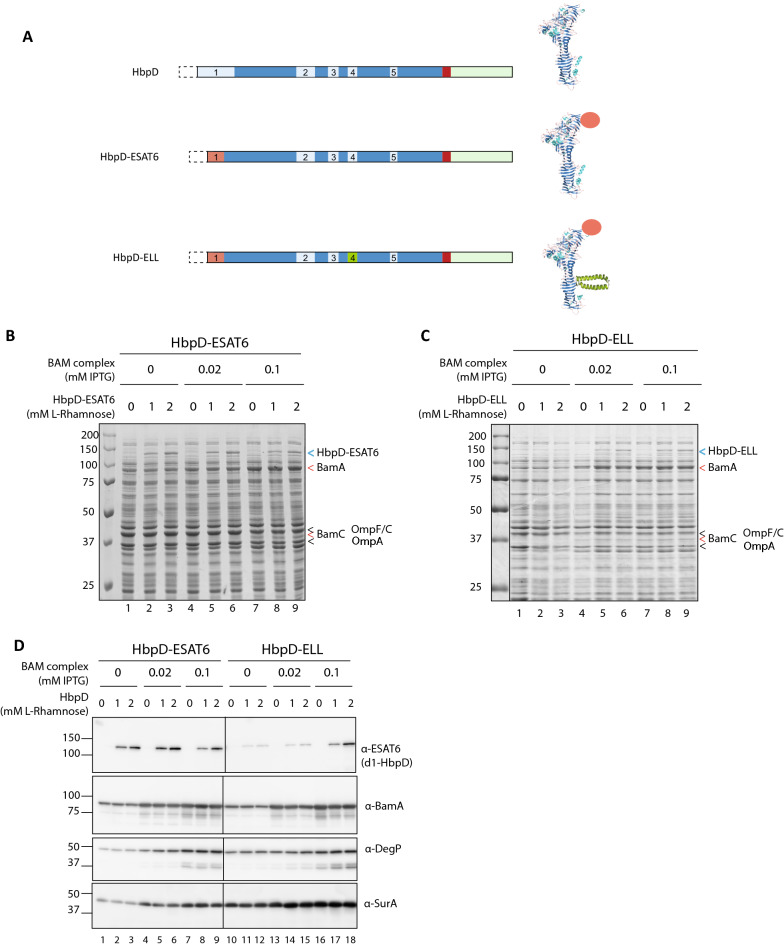
Co-overexpression of the BAM complex improved expression of HbpD-ELL. **A** Schematic representation of HbpD, HbpD-ESAT6 and HbpD-ELL with models of the folded passengers indicating the disulfide-bonded L9L9 hairpin blocking secretion. Indicated are further the mutated cleavage site in between the passenger and β-barrel domain (red), the N-terminal signal peptide (dotted box), the passenger domain (blue), and the C-terminal β-domain (pale green). Boxes d1 to d5 (light blue) represent regions of Hbp that can be exchanged for heterologous protein segments [18]. In HbpD-ESAT6, mycobacterial antigen ESAT6 (orange) replaces d1, in HbpD-ELL the L9L9 hairpin replaces d4 (green). **B**,** C** Coomassie-stained SDS-PAGE gels of whole cell lysates from *E. coli* BL21 (DE3) cultures expressing HbpD-ESAT6 (**B**) or HbpD-ELL (**C**), both with or without induction for extra BAM complex. The final concentration of inducers IPTG (BAM) and Rhamnose (HbpD) are indicated above the gel-images. The protein bands representing BamA, BamC, OmpF/C, OmpA, HbpD-ESAT6 and HbpD-ELL are indicated by “<”. **D** Western blots incubated with antisera recognizing ESAT6, BamA, DegP and SurA

For many biotechnology applications, including recombinant protein production or vaccine development, it is considered an advantage to secrete proteins into the extra-cellular milieu or to display them on the surface of the producing bacteria [18, 19, 36, 54]. Gram-negative bacteria have a complex cell envelope that consists of a cytoplasmic membrane and an outer membrane (OM) enclosing the periplasmic space with the peptidoglycan layer. Multiple secretion pathways have evolved to transport proteins across the two membranes [3]. Most pathways require multi-protein secretion complexes that span the cell envelope. By contrast, the five subclasses of the type V (or autotransporter) secretion pathway (type Va–Ve) are fairly simple and encoded by one or two genes [8]. The secreted proteins of these systems cross the cell envelope in two consecutive steps. The type V systems, in general, comprise only a single gene encoding a signal peptide at the N-terminus, a secreted protein domain often called the passenger and a β-barrel domain involved in transport of the passenger across the outer membrane [8, 30, 54]. An exception is formed by the Two Partner secretion systems, that are classified as type Vb and consist of two genes encoding the secreted protein and a dedicated transporter. Furthermore, while the passengers of the type Vc–Ve subclasses remain attached to their β-barrel domains, most passengers of the type Va subclass, the classical autotransporters, are proteolytically cleaved from their β-barrel domain after which they are released into the extra-cellular milieu or remain bound to the cell surface via non-covalent interactions (see Fig. 1 for the gene organization in classical autotransporters). Despite their apparent simplicity, type V secretion systems secrete some of the largest proteins encoded by bacterial genomes. They function in most cases as adhesin, protease, or toxin to support bacterial virulence [30].

The signal peptide is required for targeting to and transfer across the Sec-translocon in the cytoplasmic membrane. Upon translocation into the periplasm, the signal peptide is cleaved and the mature autotransporter engages periplasmic chaperones such as SurA to maintain translocation competence and trigger targeting to the BAM complex, which functions as a generic foldase and insertase for β-barrel outer membrane proteins (OMPs) [9, 22, 32]. The interaction of the BAM complex with the β-barrel domain of autotransporters not only results in their insertion into the OM. it also facilitates the transport of the passenger to the cell surface [8, 30, 54]. Recent structural studies show that the central component of the BAM complex, the integral OMP BamA, interacts with nascent OMPs to aid β-barrel folding [27, 49]. Similarly, contacts were found between BamA and the β-domain and passenger of the classical autotransporter EspP while being translocated across the OM, indicating a intricate contact between BamA and the nascent autotransporter during this process [6]. In addition to BamA, the BAM complex of *Escherichia coli* and other gammaproteobacteria consists of four accessory lipoproteins (BamB–E), although variations in composition have been found in other bacterial classes [56].

The classical autotransporters are widely applied for secretion and cell-surface display of heterologous proteins, e.g. enzymes for biotechnical applications or antigens for vaccine development [19, 50, 54]. We have studied the structure and secretion mechanism of the *Escherichia coli* autotransporter hemoglobin protease (Hbp), which helped to develop a vaccine platform in which multiple antigens are displayed at the surface of non-pathogenic bacteria and outer membrane vesicles (OMVs) [5, 13, 18, 24, 42]. Sequences encoding the antigen of choice were inserted into *hbp*, replacing those encoding subdomains of the Hbp passenger that extend from the β-helical stem that this passenger forms [13, 18, 33]. To expose such antigens on the surface of the bacterial cell or OMVs, Hbp-Display constructs (HbpD) were made that lack the autocatalytic cleavage site thus preventing release of the passenger into the extracellular milieu [18]. However, the translocation capacity of HbpD appeared to be limited by the number, size and in particular the structural complexity of the fused cargo antigens [13, 16, 41].

Previously, we have attempted to expand the secretion capacity of the Hbp system by testing adaptations to the β-barrel domain and by replacing it for the β-barrel domain of another autotransporter [14, 39] but with limited success. Here we examined whether increased expression of generic components of the secretion route of autotransporters can improve secretion of overproduced and difficult-to-secrete (DTS) recombinant Hbp chimeras. During secretion Hbp has been shown to interact with the periplasmic chaperone SurA and the BamA and BamB components of the BAM complex [40], whereas Hbp constructs that are blocked in secretion are degraded by DegP, a periplasmic protease upregulated upon cell-envelope stress [16, 47]. In addition, the periplasmic chaperones SurA and Skp and BAM complex subunits BamA, BamB and BamD with *E. coli* autotransporter EspP have been reported [11, 12]. Here, we investigated the possibility that the limited availability of these factors is a bottleneck in the secretion of DTS HbpD chimeras. Indeed, we found that overproduction of the BAM complex markedly improves surface display of such Hbp chimeras. Co-expression of various chaperones also had a positive albeit less general influence on display efficiency.

## Results

### Co-overexpression of the BAM complex improved expression of HbpD-ELL

In earlier studies, we have reported on the limited tolerance of Hbp to transport folded heterologous protein domains across the outer membrane where such DTS constructs showed a decreased secretion efficiency when compared to wild-type Hbp or efficiently-secreted derivatives [13, 16, 41]. Examples of DTS inserts are the calmodulin domain that forms a stable fold in the presence of calcium ions [16] and a single-chain antibody domain that includes two disulfide bonds [14]. To systematically investigate the limits of Hbp secretion, we previously constructed a DTS Hbp with an α-helical hairpin formed by two stable α-helices derived from ribosomal protein L9 of *Bacillus stearothermophilus* constricted into an hairpin through a disulfide bond formed between cysteine residues engineered at positions 707 and 712 of the Hbp passenger (Fig. 1A, Table 1) [41]. This model passenger was shown to be stalled during translocation across the outer membrane unless the formation of the disulfide bond was prevented [41]. This indicated that single α-helices, but not helical hairpins, are compatible with secretion. Introduction of the ESAT6 antigen from *Mycobacterium tuberculosis* at the N-terminus of the Hbp passenger, where it replaces the protease subdomain of wild-type Hbp (Fig. 1, region 1), enabled detection of the stalled construct using anti-ESAT6 monoclonal antibodies. Here we have used an uncleaved display variant of this stalled Hbp construct called HbpD-ELL (Fig. 1A). As a control served HbpD-ESAT6 (Fig. 1A), which lacks the helical hairpin and is efficiently secreted to the cell surface [18].

**Table 1:** Autotransporter Display constructs used in this study

Name	Detection antigen	Secretion blocking structure	Cell surface labelling
HbpD-ELL	ESAT6	S–S bonded L9L9 hairpin	α-ESAT6
HbpD-ESAT6	ESAT6	–	α-ESAT6
HbpD-SpT2-LL	–	S–S bonded L9L9 hairpin	SpC2-mScarlet
HbpD-SpT2	–	–	SpC2-mScarlet
HbpD-SpT2-Calm	HA tag	Calmodulin/Ca^2+^	SpC2-mScarlet
HbpD-SpT2-GFPnb	HA tag	S–S bonded GFP nanobody	SpC2-mScarlet
UpaG-SpT2-Calm	HA tag	Calmodulin/Ca^2+^	SpC2-mScarlet
UpaG-GFPnb	HA tag	S–S bonded GFP nanobody	GFP

Since the BAM complex was shown to be directly involved in the translocation of Hbp across the outer membrane [40], we considered the possibility that the endogenous level of BAM-complexes is a limiting factor for efficient secretion of DTS HbpD constructs. To relieve this potential bottleneck, we co-overexpressed the *bam* operon with either HbpD-ELL or efficiently-secreted HbpD-ESAT6 in *E. coli* BL21 (DE3). Plasmid pJH114 carries the five genes (*bamA*–*bamE*) encoding the BAM complex under control of a single IPTG-inducible *trc* promoter [37]. To have separately inducible *hbp* genes, we cloned *hbpD-ELL* and *hbpD-ESAT6* downstream of a rhamnose-inducible promoter (P*rha*) in the pLEMO plasmid [55], which is compatible with pJH114 (Table 2). Expression of the HbpD fusions with and without overexpression of the BAM complex was then compared. To ensure the availability of the additional copies of BAM for HbpD secretion, expression of the *bam* operon was induced one hour prior to induction of HbpD expression for two hours. Samples were taken and analyzed by SDS-PAGE followed by staining with Coomassie Brilliant Blue or Western blotting (Fig. 1).

**Table 2:** Plasmids used in this study

Name	*ori*	Inducible promoter	Inducer	Selection marker	References
pJH114	pMB1	P*trc*	IPTG	*bla*	[37]
pLEMO-HbpD-ESAT6	p15a	P*rha*	Rhamnose	*cat*	This study
pLEMO-HbpD-ELL	p15a	P*rha*	Rhamnose	*cat*	This study
pLEMO-HbpD-SpT2	p15a	P*rha*	Rhamnose	*cat*	This study
pLEMO-HbpD-SpT2-LL	p15a	P*rha*	Rhamnose	*cat*	This study
pLEMO-HbpD-SpT2-Calm	p15a	P*rha*	Rhamnose	*cat*	This study
pLEMO-HbpD-GFPnb	p15a	P*rha*	Rhamnose	*cat*	This study
pLEMO-UpaG-SpT2-Calm	p15a	P*rha*	Rhamnose	*cat*	This study
pLEMO-UpaG-GFPnb	p15a	P*rha*	rhamnose	*cat*	This study
pRha-HbpD-ELL	pMB1	P*rha*	Rhamnose	*kan*	This study
pRha-HbpD-SpT2-Calm	pMB1	P*rha*	Rhamnose	*kan*	This study
pRha-HbpD-GFPnb	pMB1	P*rha*	Rhamnose	*kan*	This study
pTUM4	p15a	P*cat*	Constitutive	*cat*	[44]
pTUM2	p15a	P*cat*	Constitutive	*cat*	This study
pTUM6	p15a	P*cat*	Constitutive	*cat*	This study
pET28-SpC2-mScarlet	pMB1	P*T7*	IPTG	*kan*	*Lab collection*
pET20b-GFP-His6	pMB1	P*T7*	IPTG	*bla*	J.W. de Gier, Stockholm, Sweden
pEH3-GFP	pMB1	P*lac*UV5	IPTG	*cat*	*Lab collection*

Expression of HbpD-ELL in the absence of IPTG-induced *bam* expression resulted in a clear growth defect (Additional file 1: Fig. S1) and poor production level when compared to HbpD-ESAT6 (Fig. 1, compare panels B and C), in line with earlier observations for DTS Hbp constructs [13, 41, 48]. Cultures to which no IPTG was added (so with endogenous, or near-endogenous BAM levels) expressed HbpD-ESAT6 (~ 125 kDa) upon addition of rhamnose at levels detectable both on Coomassie-stained gels and on Western blots incubated with monoclonal antiserum against ESAT6 (Fig. 1B, D), while HbpD-ELL (~ 130 kDa) was hardly detectable, likely due to stalled secretion and subsequent degradation by the periplasmic protease DegP (Fig. 1C, D [41]). Expression of HbpD-ESAT6 and HbpD-ELL by themselves induced only a moderate increase in DegP levels [16, 48] as confirmed by Western blotting (Fig. 1D). IPTG-induced production of BAM from pJH114 resulted in a dose-dependent accumulation of BamA (Fig. 1B–D) indicative of increased BAM complex production. Overexpression of BAM also clearly affected the bacterial growth rate (Additional file 1: Fig. S1) and led to a moderately increased expression of DegP (Fig. 1D, lanes 4, 7, 13, 16), while the combined expression of the HbpD constructs and BAM yielded the highest induction of DegP. Co-expression of BAM did not appear to change the expression of HbpD-ESAT6 (Fig. 1B, D), indicating that optimal levels of this efficiently secreted construct were already reached in the presence of endogenous BAM. Importantly, however, production of HbpD-ELL markedly increased upon induction of the BAM complex from pJH114 (Fig. 1C, D), suggesting a favorable effect of extra BAM on the biogenesis of this difficult to secrete HbpD variant as hypothesized.

### Co-overexpression of the BAM complex improved surface display of HbpD-ELL and HbpD-SpT2-LL

**Fig. 2:**
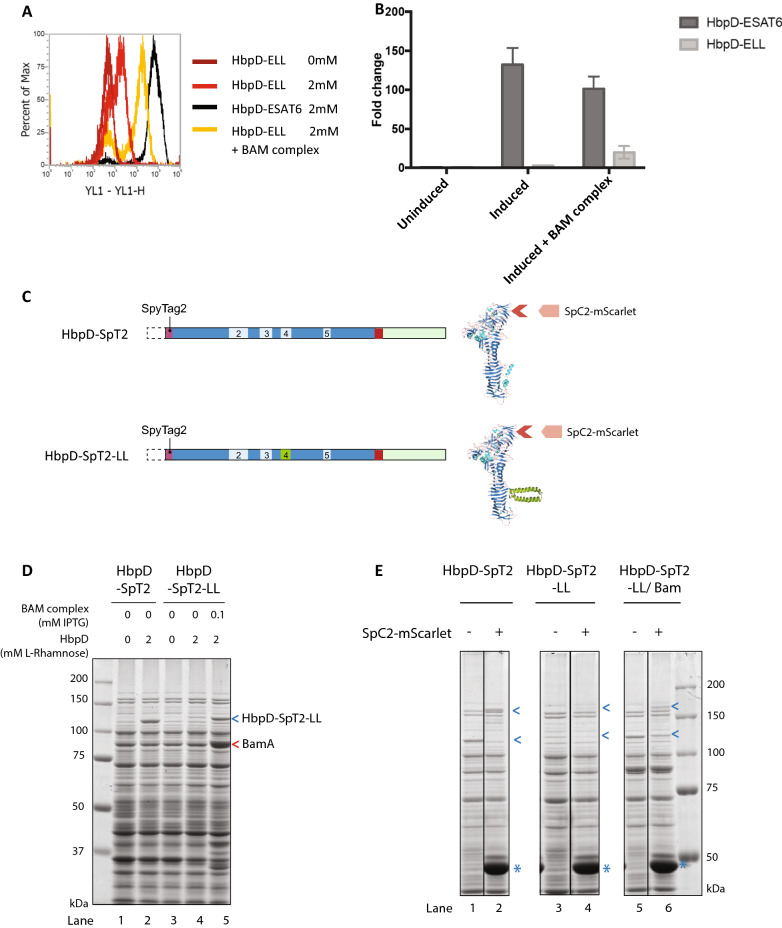
Co-overexpression of BAM improves surface display of HbpD-ELL. **A** Flow cytometric data showing the fluorescent intensity of cells bound by anti-ESAT6 monoclonal antibodies. The different culture conditions tested (i.e. HbpD-ESAT6 or HbpD-ELL uninduced, induced, or induced for BAM as well) are indicated on the right. **B** Fold change of surface display of cells expressing HbpD constructs as in (**A**). Graph shows the mean from three independent flow cytometry experiments. **C** Schematic representation and passenger models for HbpD-SpT2 and HbpD-SpT2-LL. Coupling of SpT2 to externally added SpC2-mScarlet allows detection of surface displayed HbpD-SpT2 and HbpD-SpT2-LL. **D** Coomassie-stained SDS-PAGE gels of whole cell lysates from *E. coli* BL21 (DE3) expressing HbpD-SpT2-LL with or without co-expression of BAM compared to lysates of cells expressing HbpD-SpT2 only. **E** Coomassie-stained SDS-PAGE gels of whole cell lysates from *E. coli* BL21 (DE3), expressing HbpD-SpT2 and HbpD-SpT2-LL and incubated with SpC2-mScarlet for 21 h at 4 °C. HbpD variants and SpC2-mScarlet adducts (<), BamA (<) and non-bound SpC2-mScarlet(*) are indicated. Panel **E** is composed of lanes taken from the same image of a Coomassie-stained SDS-PAGE gel; the assembled parts are boxed

We next investigated whether the increased level of HbpD-ELL in the presence of overproduced BAM complex also reflects an increase in outer membrane translocation and, consequently, surface display of this construct. Display of HbpD-ELL and HbpD-ESAT6 was first assessed by direct whole-cell immune staining using flow cytometry. Cells were grown and induced for expression of the BAM complex and the HbpD variants and analysed for surface display using anti-ESAT6 monoclonal antibodies and flow cytometry (Fig. 2A). As expected, expression of HbpD-ESAT6 resulted in a clear shift in fluorescent intensity compared to non-induced cells. Co-overexpression of BAM did not further increase the fluorescent intensity, consistent with the Western blot analysis (Fig. 1). Cells only induced for HbpD-ELL expression resulted in a far less pronounced shift in fluorescent intensity (Fig. 2A). Cells induced for expression of both BAM and HbpD-ELL showed a clear increase in fluorescent intensity (~ tenfold) compared to cells only induced for HbpD-ELL (Fig. 2B), indicating that increased expression of BAM, indeed, coincided with increased display of HbpD-ELL at the cell surface (Fig. 2A, B). However, as also suggested by the Western blot data, the levels of surface-displayed HbpD-ELL did not reach those of the HbpD-ESAT6 control.

To confirm that the effect of *bam* co-overexpression on surface display was independent of antibody-based labelling, we used a direct labelling method that exploits the recently developed SpyTag/SpyCatcher protein ligation system [57]. In this system, a Spy-catcher domain (SpC2) interacts with a Spy-tag peptide (SpT2) to form a covalent isopeptide bond. We exchanged the ESAT6 antigen in HbpD-ELL and HbpD-ESAT6 for SpT2, resulting in HbpD-SpT2-LL and HbpD-SpT2, respectively (Fig. 2C, Table 1). The constructs were cloned in a pLEMO plasmid under the control of P*rha*. Moreover, we fused the SpC2 domain to fluorescent protein mScarlet [1] yielding SpC2-mScarlet, which can be coupled to HbpD-SpT2 constructs displayed at the cell surface. As expected, DTS HbpD-SpT2-LL (121 kDa) was hardly detectable in BL21 (DE3) (Fig. 2D, lane 4) and detection improved upon co-expression of BAM (Fig. 2D, lane 5). To detect cell-surface exposure of HbpD-SpT2-LL and HbpD-SpT2, intact cells were incubated with purified SpC2-mScarlet. Analysis by SDS-PAGE revealed a complete shift of the band representing HbpD-SpT2 to a higher position in the gel, suggesting that most of the protein was available for coupling and, hence, exposed at the cell surface (Fig. 2E, lanes 1–2). HbpD-SpT2-LL showed a similar shift in gel position when incubated with SpC2-mScarlet, again indicative of cell-surface exposure (Fig. 2E, lanes 3–6). However, and in contrast to HbpD-SpT2, not all HbpD-SpT2-LL expressed in presence of overproduced BAM shifted, indicating that not all HbpD-SpT2-LL was displayed at the cell surface. To verify that the shift in gel position was caused by coupling to SpC2-mScarlet we analyzed the samples by semi-native PAGE and imaged the gel for fluorescent protein bands. This confirmed the presence of fluorescent SpC2–mScarlet-containing adducts coupled to HbpD-SpT2-LL (Additional file 1: Fig. S2). In conclusion, co-overexpression of the BAM-complex resulted in improved expression and display of DTS HbpD-ELL and HbpD-SpT2-LL fusions at the cell-surface.

### Overexpression of the BAM complex improves secretion of two other DTS HbpD constructs

**Fig. 3:**
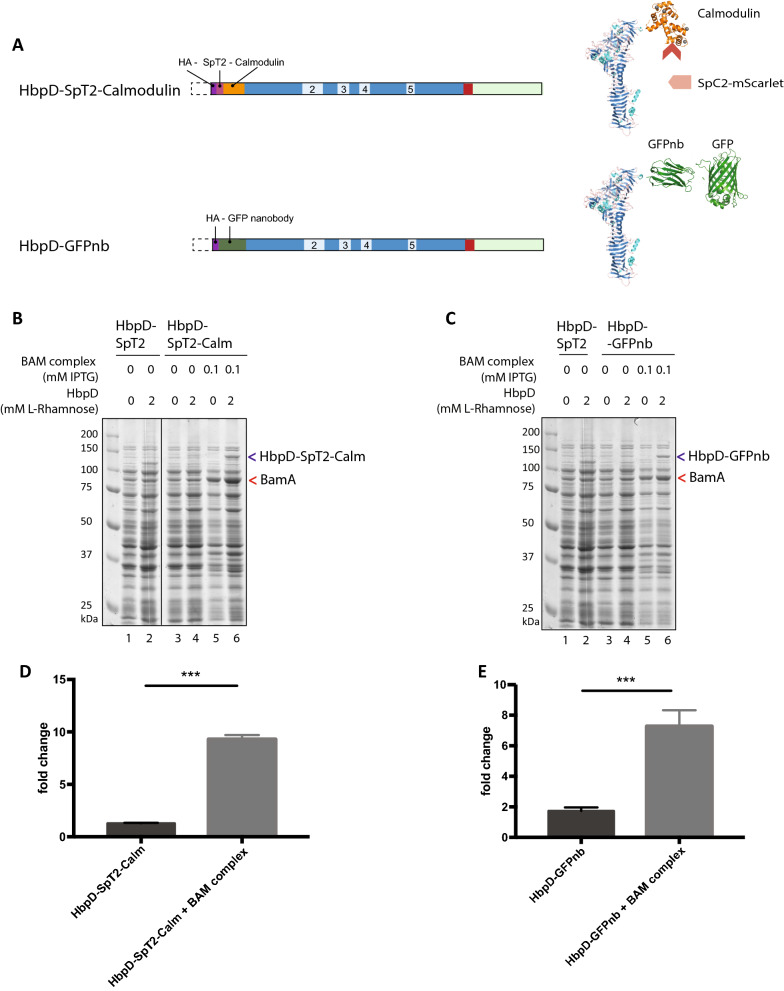
Co-overexpression of BAM improved the secretion of HbpD-Calm or HbpD-GFPnb chimeras. **A** Schematic representation and passenger models for HbpD-SpT2-Calm and HbpD-GFPnb. Coupling of SpC2-mScarlet allows detection of surface displayed HbpD-Spt2-Calm fusions, while binding of GFP to the GFPnb allows the detection of surface displayed HbpD-GFPnb. **B**, **C** Coomassie-stained SDS-PAGE gels with whole cell lysates of *E. coli* BL21 (DE3) cultures expressing HbpD chimeras. **B** Expression of HbpD-SpT2-Calm with and without overexpression of BAM compared to HbpD-SpT2. **C** Expression of HbpD-GFPnb with and without overexpression of BAM complex compared to HbpD-SpT2. **D**, **E** Fold change of fluorescence as detected by flow cytometry for binding of SpC2-mScarlet (**D**) or GFP (**E**) to HbpD-SpT2-Calm and HbpD-GFPnb when expressed with or without overproduced BAM complex. ***p < 0.005

Our results indicated a clear and positive effect of extra copies of the BAM complex on the expression and display of HbpD-ELL and HbpD-SpT2-LL. To investigate whether this is a generic effect, we tested the influence of co-expression of BAM on DTS HbpD constructs that carry either a calmodulin or a single-chain antibody domain. These segments were inserted at the N-terminus of the passenger, where they replaced the protease subdomain of Hbp (Fig. 3; Table 1).

Calmodulin is a well-characterized domain of 145 amino acids (without any cysteines) that folds into a stable tertiary conformation in presence of Ca^2+^ ions [16, 58]. The N-terminus of the calmodulin construct was extended with SpT2 and HA tags, yielding HbpD-SpT2-Calm (Fig. 3A) and cloned into the pLEMO plasmid. Subsequently, *E. coli* BL21 (DE3) already carrying BAM-expression plasmid pJH114 was transformed with the pLEMO vector encoding HbpD-SpT2-Calm or HbpD-SpT2 as control. The resulting co-transformants were grown in medium supplemented with Ca^2+^ to promote calmodulin folding and the same induction scheme was applied as outlined for BAM and HbpD-ELL above. Analysis of the samples in which BAM was not induced resulted in detection of the HbpD-SpT2 at ~ 117 kDa upon, whereas DTS HbpD-SpT2-Calm migrated as a faint band at ~ 138 kDa, suggesting inefficient secretion and concomitant degradation (Fig. 3B, lanes 2 and 4). However, BAM overexpression resulted in increased detection of HbpD-SpT2-Calm, reaching the level observed for the positive control HbpD-SpT2 (Fig. 3B, lane 6). To examine whether the improved expression levels coincided with improved cell-surface display, cells were incubated with SpC2-mScarlet and analyzed by flow cytometry (Fig. 3D). The results showed a low level of coupling of SpC2-mScarlet to un-induced cells and cells induced only for HbpD-SpT2-Calm. In contrast, co-expression of HbpD-SpT2-Calm with BAM led to an eight-fold increase of SpC2-mScarlet coupling, indicative of improved cell-surface display.

We then tested HbpD fused to a nanobody that binds GFP (GFPnb; Fig. 3A) [52]. Nanobodies are the antigen-binding fragments of the light chain of IgG antibodies (also known as V_HH_). These small domains (15 kDa) are known to fold in the periplasm and include one disulphide bond. Fusing GFPnb to HbpD has been shown to impair expression and surface display [14, 52]. We tested the effect of co-expression of BAM on expression and display of DTS HbpD-GFPnb cloned into pLEMO. Cells not induced for extra BAM showed low expression levels of HbpD-GFPnb, when compared to the positive control HbpD-SpT2 (Fig. 3C, lane 2 and 4). However, expression of HbpD-GFPnb improved considerably in cells co-expressing BAM (Fig. 3C, lane 6). Improved cell-surface display was shown by incubating the cells with purified GFP followed by flow cytometry, which showed a clear shift of the fluorescence peak when BAM was over-expressed (Fig. 3E).

Taken together, overproduction of the BAM complex improves expression and surface display of three DTS variants, suggesting that BAM overexpression improves the tolerance of Hbp secretion for complex domains.

### Overexpression of the BAM complex supports display of a DTS trimeric autotransporter construct

**Fig. 4:**
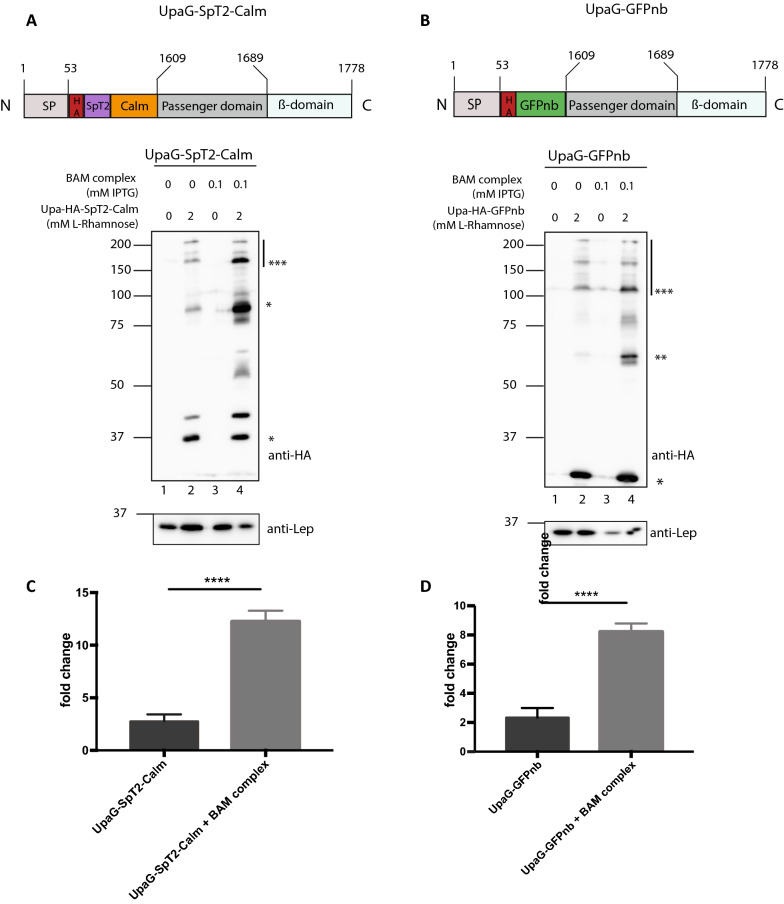
Co-overexpression of BAM improves surface display of DTS trimeric autotransporter UpaG chimeras. **A**, **B** Schematic representation of UpaG-SpT2-Calm (**A**) and UpaG-GFPnb (**B**) and Western blots of whole cell lysates to analyze their expression in BL21 (DE3). The HA tag, SpT2 tag and calmodulin domains in (**A**) and GFPnb in (**B**) were inserted at the N-terminus of the truncated passenger domain (UpaG position are given). The UpAG bands were detected by incubating the Westernblots with anti-HA. The putative positions of monomeric (*), dimeric (**) and trimeric (***) complexes [45] are indicated on the right side of the panels. **C** Fold change of surface displayed UpaG- -SpT2-Calm, as indicated by flow-cytometric analysis of binding of SpC2-mScarlet, when expressed with or without overproduced BAM. **D** Fold change of surface displayed UpaG-GFPnb, as indicated by flow cytometric analysis of binding of GFP, when expressed with or without overproduced BAM. ***p < 0.005. Data presented is the mean of three independent experiments

Not only the classical autotransporters, but also the other single-gene type V subclasses (Vc–Ve) require the BAM complex for translocation of their passengers to the cell surface [8]. We, therefore, investigated the effect of BAM overexpression on two DTS chimeras of a trimeric autotransporter (type Vc). Trimeric autotransporters share the domain organization of monomeric autotransporters, but their β-barrel and passenger domains assemble in trimers. We used a 170-residue truncate of UpaG, a trimeric autotransporter adhesin and known virulence factor of pathogenic *E. coli* [45, 51]. We designed two potentially DTS UpaG variants with N-terminally either SpT2-Calmodulin (UpaG-SpT2-Calm; Fig. 4A; Table 1) or GFPnb (UpaG-GFPnb; Fig. 4B; Table 1). The pLEMO plasmids carrying these constructs were introduced in *E. coli* BL21 (DE3) harboring pJH114 and expression was tested as before.

When expression of the BAM complex was not induced, the two UpaG constructs were expressed to levels detectable on Western blot showing bands at ~ 35 kDa and ~ 30 kDa corresponding to the predicted masses calculated for monomeric UpaG-SpT2-Calm (38 kDa) and UpaG-GFPnb (32 kDa), respectively (Fig. 4A, B lane 2). Additional bands at higher positions in the gel could represent multimeric forms of the constructs, with the stronger reacting bands likely representing dimeric and multiple conformations of the trimeric form (Fig. 4A, B). Such a pattern was observed earlier for truncated UpaG [45] and trimeric autotransporter YadA [2]. Of note, trimeric autotransporters are known to be resistant to denaturation in SDS-PAGE [4]. Interestingly, expression of BAM from pJH114 together with the two DTS UpaG derivatives resulted in an increased intensity on gel of those bands that presumably represent dimeric and trimeric forms (Fig. 4A, B; lanes 4). Subsequent analysis by flow cytometry showed that co-expression of BAM resulted also in improved cell-surface exposure of UpaG-SpT2-Calm and UpaG-GFPnb (Fig. 4C, D). The addition of SpC2-mScarlet (Fig. 4C) or GFP (Fig. 4D) to cells co-expressing BAM and the two UpaG constructs yielded a significant increase in fluorescent signal compared to cells only induced for the UpaG constructs or non-induced cells. Apparently, the over-produced BAM complex improved cell surface display of both UpaG derivatives, suggesting that this was a rate-limiting step in the process. Overall, we conclude that the effect of BAM overproduction is likely generic for type V secretion systems.

### The influence of overproduction of periplasmic chaperones on DTS HbpD constructs

**Fig. 5:**
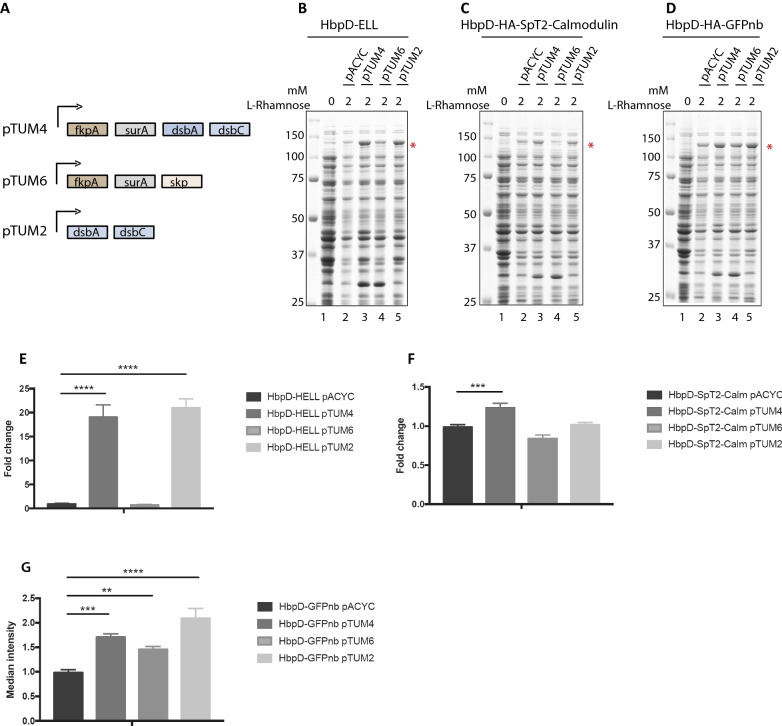
The influence of overproduction of periplasmic chaperones on difficult-to secrete HbpD constructs. **A** Schematic representation of pTUM plasmids used in this study indicating the different combinations of chaperones (FkpA, SurA and Skp) and folding catalysts (DsbA and DsbC) used. The plasmids are pACYC derivatives, which was used as negative control. **B**–**D** Coomassie-stained SDS-PAGE gels showing expression of DTS HbpD constructs in combination with the pTUM plasmids. Tested were HbpD-ELL (**B**), HbpD-SpT2-Calm (**C**) and HbpD-GFPnb (**D**). **E**–**G** Fold change of surface displayed HbpD constructs when co-expressed with periplasmic chaperones, as shown by flow cytometry using anti-ESAT6 (**E**), coupling of SpC2-mScarlet to SpT2 (**F**) and binding of GFP to the HbpD-GFPnb (**G**). **p < 0.01; ***p < 0.005; ****p < 0.001

Chaperones like DsbA, Skp, SurA and DegP keep secretion intermediates of autotransporters in a translocation competent state during their transit of the periplasm [54]. We, therefore, probed the effect of overproduction of periplasmic chaperones on the surface localization of the three DTS HbpD variants analysed here. Previous findings by the Skerra lab revealed that a combination of DsbA, DsbC, FkpA, Skp and SurA enhanced the expression of disulphide-bond containing proteins in *E. coli* [43]. DsbA and DsbC are thiol-disulfide oxidoreductases that catalyze the formation of disulfide bonds [25], FkpA functions as a general folding enhancer, while Skp and SurA are chaperones that escort β-barrel OMPs, including autotransporters, to the BAM complex [10, 23, 26]. To test the effect of extra copies of these chaperones on DTS HbpD constructs, we co-transformed BL21 (DE3) with a pTUM plasmid encoding chaperones and a pRHA plasmid encoding either HbpD-ELL, HbpD-SpT2-Calm, or HbpD-GFPnb (Table 1). The pTUM plasmids used were pTUM2 (encoding DsbA and DsbC), pTUM4 (encoding DsbA, DsbC, FkpA and SurA) and pTUM6 (encoding FkpA, SurA and Skp). While pTUM6 did not seem to have a highly significant beneficial effect, pTUM4 or pTUM2 improved expression of all three DTS HbpD derivatives albeit to different extents (Fig. 5B–D, lanes 3–5). The improved expression also led to an increase in cell surface display, as detected by flow cytometry (Fig. 5E–G). The fact that both pTUM2 and pTUM4 improved the expression of HbpD-ELL suggested that DsbA and DsbC affected the disulphide bond that is instrumental in blocking HbpD-ELL secretion [41]. On the other hand, presence of pTUM2 and pTUM4 also improved binding of SpC2-mScarlet to HbpD-SpT2-Calm and of GFP to HbpD-GFPnb (Fig. 5F, G), albeit with far lower fold-changes. The results are consistent with the reported effect of pTUM4 on expression of periplasmic proteins that lack disulfide bonds [44], and, therefore, may suggest that the slightly increased surface localization of DTS constructs tested here might be a direct or indirect consequence of the chaperones involved.

## Discussion

Autotransporter-mediated surface display (also referred to as autodisplay) has been used in a variety of biotechnical applications including vaccine development, whole-cell biocatalysis, biosensor development, epitope mapping, and peptide library screening [15, 19, 31]. However, secretion of chimeric autotransporter constructs is limited by the size, structural complexity and folding propensity of the inserted cargo. In this study, we show that the secretion of recombinant DTS autotransporter chimeras can be improved by increasing the cellular concentration of the BAM complex and periplasmic chaperones. We opted to investigate these factors since our work had shown they associate with DTS derivatives of the model autotransporter Hbp that are jammed in the outer membrane due to engineered disulfide bonds or to insertion of folded protein segments [14, 16, 40]. Similarly, a mutation in the C-terminal region of the passenger that is thought to nucleate its folding at the cell surface resulted in a stalled OM intermediate [47]. Finally, RNA sequencing showed that accumulation of a DTS Hbp mutant led to increased levels of mRNA encoding subunits of the BAM complex [48].

A plausible explanation for the beneficial effect of BAM overexpression is that translocation and OM-insertion of complex HbpD derivatives is slow, thus titrating available BAM complexes. This would trigger degradation in the periplasm by DegP and other proteases involved in cell envelope quality control [16, 48]. Indeed, DTS Hbp derivatives induce extra-cellular stress responses and higher levels of the DegP proteases. BAM overexpression may provide more assembly sites for translocation but may also shield Hbp derivatives not yet at the cell surface from degradation in the periplasm. The latter explanation may be supported by the observation that externally adding SpC2-mScarlet to cells expressing DTS Hbp-SpT2 constructs did not result in full binding of these HbpD-derivatives, while near-complete binding was observed for HbpD-SpT2 (Fig. 2). Furthermore, overexpression of BAM did not improve secretion of non-blocked variants of HbpD, like HbpD-ESAT6 or HbpD-SpT2, implying maximally efficient expression at endogenous levels of BAM. Similarly, increased expression of BAM did not influence the levels of other β-barrel OMPs, such as OmpA and the major porins OmpF and OmpC. A negative trade-off of co-expression of the BAM complex is a reduction in growth of the cultures (Additional file 1: Fig. S1) and an increase of cell-envelope stress as judged by the increase of DegP and SurA levels in cells when BAM is overexpressed (Fig. 1D). Nevertheless, vital processes, like Sec-mediated translocation across the cytoplasmic membrane, appeared not affected much, as indicated by the absence of accumulation of unprocessed SurA, a soluble periplasmic protein transported by the Sec translocon (Fig. 1D).

We initially focused on the monomeric autotransporter Hbp, but BAM co-expression also improved the secretion of trimeric autotransporter UpaG chimeras. Possibly, other β-barrel proteins that are difficult to assemble in the outer membrane could also benefit from the presence of extra copies of the BAM complex, which may expand its use for biotechnical applications. For example, the inverse autotransporters (subclass type Ve) have also been used for surface display [38]. An alternative strategy to BAM co-expression could be to lower the amount of substrates for endogenous BAM. This has been achieved by deleting genes encoding or regulating OMPs, yielding strains that showed improved expression of recombinant OMPs and trimeric autotransporter constructs [29, 34]. However, in our hands the strain of Prilipov et al. did not yield the levels of HbpD-ELL obtained when co-expressing BAM (results not shown).

In comparison to the generic effect of co-expressing BAM, the effect of co-expressing periplasmic chaperones and folding catalysts [44] appeared variable and limited (Fig. 5). The major effect observed, a positive effect of DsbA/C on HbpD-ELL expression and display, can very likely be attributed to the release of the secretion-blocking disulphide bond in the construct [41]. Apparently, overproduction of both DsbA and DsbC results not only in increased formation of disulphide bonds, but also in increased reduction of these bonds, causing modulation between stalled and secretion-competent states. However, we cannot rule out an indirect effect of DsbA/DsbC on DTS Hbp constructs, in view of the observed effects on the chimeras that lack cysteines. In contrast, overexpression of SurA and Skp, which have been reported to interact with nascent autotransporters in the periplasm [12, 40, 47] showed only a limited influence.

Our earlier unsuccessful attempts to improve the secretion of DTS Hbp chimeras focused on modifying the β-barrel at the C-terminus. These approaches included inserting extra β-hairpins to enlarge the β-barrel channel [39], or replacing the β-barrel for the larger β-barrel of OMP FhuA (unpublished data). However, the interactions between passenger, β-barrel domain and BAM complex may be too fine-tuned to tolerate such adaptations. A simple replacement of the Hbp β-domain for that of the nearly identical autotransporter EspP already caused a reduction in secretion efficiency of the Hbp [39]. It is also clear from our work and that of many others, that the choice of β-barrel domain and the site where recombinant proteins are fused to the β-barrel or inserted within the passenger domain may influence secretion efficiency and requires testing of different possibilities [14, 35]. The results presented here suggest that co-expression of the BAM complex offers a straightforward and generic solution for impeded autotransporter secretion, probably by providing an extended time-window for the secretion process. It remains to be shown whether it is also a solution to improve the expression of other recombinant β-barrel proteins.

## Materials and methods

### Strains and growth conditions

*E. coli* strain Top 10F’ (Invitrogen, UK) was used for cloning and BL21(DE3) (Novagen, Germany) was used for expression experiments. Both were grown in lysogeny broth (LB; 10 g/L tryptone, 5 g/L yeast extract, 10 g/L NaCl). For expression experiments the LB was supplemented with 0.2% (w/v) glycerol. To select for plasmids (Table 2) antibiotics were added to the following concentrations: ampicillin, 100 µg/mL; kanamycin, 50 µg/mL; and chloramphenicol 30 µg/mL. Unless stated otherwise, cultures were incubated at 37 °C with shaking.

### Reagents, chemicals, enzymes and sera

Rapid DNA De-phosphorylation and Ligation Kit was obtained from Roche Applied Science, restriction enzymes and Phusion High Fidelity DNA polymerase from New England Biolabs. The pre-stained Precision Plus SDS-PAGE protein marker was obtained from Biorad. Sigma-Aldrich provided all other reagents, primers and chemicals. 96-well plates used were μClear Chimney black clear-bottom plates from Greiner. Immunostaining was performed with mouse monoclonal antibodies directed against HA, ESAT6 (Hyb 76-8) [21] or with rabbit polyclonal serum recognizing the Hbp β-barrel (SN477) [53], BamA (a kind gift of J. Tommassen, Utrecht University, The Netherlands), SurA (a kind gift of T. Silhavy, Princeton University, USA), leader peptidase (Lep; from our own laboratory collection), or DegP (a kind gift of J. Beckwith, Harvard Medical School, USA). Secondary antisera used were peroxidase-conjugated goat-anti-mouse and goat-anti-rabbit IgGs (Rockland Immunochemicals). Lumi-Light Western Blotting Substrate was obtained from Roche, skim milk from Thermo Fisher Scientific. All synthetic DNA constructs were obtained using GeneArt (Thermo Scientific).

### Expression plasmids

The BAM complex was expressed from plasmid pJH114 which carries the five genes *bamA–E* cloned into an operon under the control of an IPTG-inducible *trc* promoter [37]. This plasmid was co-transformed with pLEMO [55] and pRha-derived [7] plasmids (a kind gift of J.W de Gier, Stockholm University, Sweden) carrying the regulatory cassette enabling the rhamnose-mediated induction of P*rha* and the HbpD-constructs described in Table 2 under control of this promoter. All constructs have been confirmed by sequencing (Macrogen). The plasmids encoding the chimeras were constructed as follows:

#### HbpD-ESAT6 and HbpD-ELL constructs

Fragments including the ORFS encoding HbpD-ESAT6 were obtained by was produced by PCR using pEH3-HbpD-ESAT6 plasmid [18] and pEH3-HbpD-ELL [41] as a template and primers Fw-EcoRI-SalI-HbpD (5-CGAATTCGTCGACACCATGAACAGAATTTATTCTCTTCGC-3ʹ) and Rv-HbpD-BamHI-HindIII (5ʹ-CCAAGCTTGGATCCTCAGAATGAATAACGAATATTAGCG-3ʹ). The PCR products were digested with SalI and BamHI and ligated into pLemo plasmid digested with the same enzymes. The corresponding pRha-HbpD-ELL plasmid was also made by PCR using the same template and forward primer in combination with primer HindIII-Hbp-Rv (5ʹ-CTGAAAGCTTCAGAATGAATAACGAATATTAGCG-3ʹ) and cloned into the pRha vector using a SalI-HinDIII fragment.

#### HbpD-SpT2 and HbpD-SpT2-L9L9

To allow for direct labelling of Hbp chimeras at the cell surface we used the SpyTag/SpyCatcher protein ligation system [57]. In this system, a Spy-catcher domain interacts with a Spy-tag peptide to form a covalent isopeptide bond. An optimized version of the tag, SpT2 [20] was cloned into *hbp* to replace the ESAT6 antigen, resulting in HbpD-SpT2-LL and HbpD-SpT2, respectively (Fig. 2C). For cloning a synthetic DNA fragment including restriction sites SacI and EagI was ordered and subsequently cloned into pLemo-HbpD-ELL and pLemo-HbpD-ESAT6 cut with the same enzymes.

#### HbpD-SpT2-Calm and HbpD-GFPnb

A synthetic DNA fragment encoding HA-SpT2-Calm was cloned into an pEH3-HbpD construct [18] by in-fusion cloning (Invitrogen) using the SacI/BamHI restriction sites present in that plasmid resulting in pEH3-HbpD-SpT2-Calm. Next, a SacI–KpnI fragment from this plasmid was inserted into pLEMO-HbpD-ESAT6 plasmid digested with the same enzymes, resulting in pLemo-HbpD-SpT2-Calm. Following the same procedure, a synthetic DNA fragment encoding HA-GFPnb was ordered and inserted into the pLemo-HbpD-ESAT6 plasmid to yield pLemo-HbpD-GFPnb. The pRha plasmids encoding HbpD-SpT2-Calm and HbpD-GFPnb were subsequently made by replacing the SacI–KpnI fragment of pRha-HbpD-ELL by a SacI–KpnI fragment of pLemo-HbpD-SpT2-Calm and pLemo-HbpD-GFPnb, respectively.

#### UpaG-SpT2-Calm and UpaG-GFPnb

Expression of full-length (1778 residue) UpaG protein was reported to be toxic in *E. coli* K12, but a 170-residues truncate of UpaG that contains the signal peptide and ~ 80 residues of the native-passenger domain did not affect growth [46] and an N-terminal fusion of the SpT2 tag resulted in its exposure on the cell surface [45]. We used this information to design two potentially DTS variants of the truncated UpaG, UpaG-SpT2-Calm (Fig. 4A) and UpaG-GFPnb (Fig. 4B). A ~ 1.1 kb synthetic DNA encoding UpaG-SpC2 including restriction sites for EcoRI and HindIII was cloned into the HinDIII and EcoRI sites of pRha by Gibson assembly [28]. Subsequently, a 1.3 Kbp synthetic encoding HA-SpT2-Calm was cloned into the XbaI and EcoRI sites, to replace the HA-SpC2 encoding part, yielding pRha-UpaG-SpT2-Calm. Then, the ORF encoding UpaG-SpT2-Calmodulin fragment was amplified by PCR using pRha-med-UpaG-SpT2-Calm as template and primers c4424_SalI_FW (5ʹ-CGTCGACATGAACAAAATCTTCAAAGTAATCTGGAACC-3ʹ) and c4424_AvrII_Rv (5ʹ-CCTAGGTTACCACTGGATACCTGCCC-3ʹ). The PCR product was digested with SalI and AvrII and ligated into the pLemo plasmid digested with the same enzymes generating pLemo-UpaG-SpT2-Calmodulin. Similarly, a synthetic 391-bp HA-GFPnb-encoding DNA fragment was introduced into pRha-med-UpaG-SpT2-Calmodulin to replace HA-SpT2-Calmodulin using HindIII and BamHI. The ORF encoding UpaG-GFPnb was then amplified by PCR using pRha-med-UpaG-GFPnb as a template and primer pair c4424_SalI_FW and c4424_AvrII_Rv. The PCR product was then digested with SalI and AvrII to be introduced into the pLemo plasmid cut with the same enzymes generating pLemo-UpaG-GFPnb.

#### Other plasmids

The ORF encoding the fusion of SpC2 [20] and mScarlet [1] was based upon the published sequences and cloned into the NcoI and HinDIII restriction sites of the pET28a plasmid (Novagen). The ORFs encoding FkpA and SurA were deleted from pTUM4 (a kind gift of A. Skerra, Technische Universität München, Germany) to yield pTUM2, encoding the DsbA and DsbC proteins. To construct pTUM6, first the *dsbA* and *dsbC* genes were deleted from pTUM4 and the ORF encoding the Skp gene was inserted.

### Growth and protein expression in 96-well plates

Growth and expression assays were performed in 96-well plates. Bacteria were grown in regular culture flasks in LB medium containing 0.2% glycerol to mid-log phase. Subsequently, the culture was diluted to an optical density at 600 nm (OD600) of 0.2 and 200 μL was transferred to a 96-well plate and growth was continued for 1 h. First, expression of the BAM complex was induced by adding IPTG to a final concentration of 100 μM. Growth was prolonged for 1 h, followed by the induction of genes encoded on the pLemo/pRha plasmids by adding l-Rhamnose to a final concentration of 2 mM, after which growth was continued for 2 h. *During growth, p*lates were sealed and placed in Thermostar (BMG Labtech) shakers at 600 RPM. The OD600 was measured using the HTX Synergy plate-reader (BMG Labtech). After 2 h incubation with 2 mM l-Rhamnose, culture samples were taken by mixing the cultures with an equal volume of two-times concentrated sample buffer. Samples were then boiled for 10 min and run on SDS-PAGE gels. The gels were either stained with Coomassie Brilliant Blue or subjected to immune-blotting as described earlier [17].

### ESAT6-based flow cytometry

Bacterial cultures were started at an OD of 0.05. When cultures reached OD600 of 0.6, IPTG was added to a final concentration of 0.1 mM and grown for 1 h. Then, l-Rhamnose was added to a final concentration of 2 mM and incubation was prolonged for 2 h. In separate cultures, *E. coli* BL21 DE3 carrying pEH3-GFP [52] was grown and IPTG was added to a final concentration of 1 mM to induce GFP production. After induction, the cultures were grown for 2 h. After culturing, 0.02 OD600 units of *E. coli* cultures expressing the DTS constructs, or their controls, to be tested were mixed with 0.18 OD600 units of *E. coli* expressing GFP. The GFP-expressing bacteria were added to obtain a sufficient number of cells, while reducing the amount of anti-ESAT6 antiserum needed for efficient labeling. The mixtures were harvested by centrifugation at 4 °C and 9000×*g* for 5 min., washed once with PBS (pH 7.4) and then resuspended in PBS containing 0.7% poly-formaldehyde (PFA) and fixed overnight at 4 °C. PFA was washed away by centrifuging two times, resuspending the fixed cells in PBS. After this, pellets were resuspended in 50 µL PBS 1% BSA containing a 1:25 dilution of ESAT6 (Hyb 76–8), incubated for 1 h at room temperature, followed by 3 times of washing with 750 µL PBS 1% BSA/0.05% Tween20. Next, the pellets were resuspended in 50 µL PBS 1% BSA containing a 1:50 dilution of the Goat anti-mouse antiserum conjugated to Alexa Dye 568 and 1:1000 dilution of SYTO63 to stain the bacterial DNA. After incubation for 1 h at room temperature, the samples were washed three times with 750 µL PBS 1% BSA/0.05% Tween20. Finally, pellets were resuspended in 1 mL PBS 1% BSA and subjected to flow-cytometric analysis on an Attune NxT flow cytometer (ThermoFisher). Bacteria were gated based on the DNA stain SYTO63 (RL1-670/14 nm). GFP expression was detected in gate BL1-530/30 nm and ESAT6 (through antibody-bound Alexa Dye 568) was detected in YL1-585/16 nm. The fluorescence on the cells of interest (SYTO63+, GFP−) was quantified and compared to cells stained for secondary antibody only.

### Purification of SpC2-mScarlet and GFP-His6

*E. coli* BL21(DE3) cells harboring pET28-SpC2-mScarlet or pET20b-GFP-His6 were grown in LB containing 0.2% glucose to an OD600 of 0.4–0.5. IPTG was added to a final concentration of 1 mM, after which cultures were grown for another 2–4 h. Cells were harvested by centrifugation, washed with PBS (pH 7.4) then resuspended in buffer A (50 mM Na_3_PO_4_, 300 mM NaCl [pH 7.4]). Phenylmethylsulfonyl fluoride (PMSF) was added to a concentration of 125 µM. The cells were then disrupted by two passages through a One Shot cell disruptor (Constant Systems Ltd., UK) set at 1.2 Pa. Cell debris was removed by centrifugation at 4 °C and 10,000×*g* and membrane fragments were removed by centrifugation at 4 °C and 293,000×*g*, respectively. The His6-tagged proteins were then purified from the supernatant using Talon Superflow medium (GE Healthcare Life Sciences) according to the manufacturer’s instructions. Eluates were dialyzed overnight at 4 °C against up to 1000 volumes of PBS (pH 7.4). After dialysis, glycerol was added to 10%, and aliquots were stored at − 80 °C.

### Spy ligation of SpC2-mScarlet in cells

To 0.5 OD600 units of *E. coli* BL21 DE3 cells expressing either the BAM complex, the SpT2 HbpD constructs, or both, 5 μL of purified SpC2-mscarlet (9.2 mg/mL) was added. The mixtures were incubated overnight at 4 °C to allow protein ligation to occur. The cells were harvested by centrifugation and washed with PBS containing 0.05% Tween 20 and fixed in 2% PFA in PBS for 15 min, followed by three times of washing with 750 μL PBS containing 0.05% Tween 20. The mixtures were then incubated with Syto62 (1:1000 dilution) for 10 min, washed with PBS containing 0.05% Tween 20 and analyzed by flow-cytometric analysis as described above. For SDS-PAGE, equal volumes of cells were mixed with 2× sample buffer and boiled for gels stained by Coomassie Brilliant blue. To analyse fluorescence, semi-native SDS-PAGE was performed, by omitting the SDS was omitted from the gels, but not from the running buffer and loading of non-boiled samples. After electrophoresis, the gels were immediately imaged for GFP fluorescence using an AI600 imager (Amersham).

### GFP binding to GFPnb on cell surface

To 0.5 OD of *E. coli* BL21 DE3 strains expressing either the BAM complex, the DTS GFPnb constructs, or both 1.63 μL of purified GFP (92 µM) was added. After 15 min of incubation on ice, the mixtures were spin down for 5 min at 5000 rpm and washed 3 times with PBS containing 0.05% Tween 20. Next, the mixtures were incubated with Syto9 (1:1000 dilution) for 10 min, then washed with PBS containing 0.05% Tween 20 and analyzed by flow-cytometric analysis. Samples were also mixed with 2× sample buffer and subjected to SDS-PAGE and staining with Coomassie Brilliant blue. To analyse fluorescence, semi-native SDS-PAGE was performed as described above.

## Supplementary Information

**Additional file 1: Figure S1.** Plot of the growth curves of BL21 DE3 expressing either HbpD-ESAT6, HbpD-ELL alone or together with BAM from pJH114. **Figure S2.** Fluorescence image of a semi-native SDS-PAGE gel with samples of cultures co-expressing HbpD-SpT2, HbpD-SpT2-LL or HbpD-SpT2-LL with BAM that were incubated with SpC2-mScarlet or not to allow for coupling of the fluorescent protein to the HbpD-SpT2 variants exposed on the cell surface.

## Abbreviations

BAM: β-Barrel assembly machinery
OM: Outer membrane
OMP: Outer membrane protein
Hbp: Hemoglobin protease
OMVs: Outer membrane vesicles
HbpD: Hbp Display constructs
DTS: Difficult-to-secrete
IPTG: Isopropyl β-d-1-thiogalactopyranoside
Calm: Calmodulin
SpT2: Spy tag 2
SpC2: Spy catcher 2
HA: Haemagglutinin
GFP: Green fluorescent protein
GFPnb: GFP-recognizing nanobody
PMSF: Phenylmethylsulfonyl
