# Supplementary material for: Overproducing the BAM complex improves secretion of difficult-to-secrete recombinant autotransporter chimeras
Source: Microb Cell Fact. 2021 Sep 6;20:176. doi: 10.1186/s12934-021-01668-2 (PMC8419823; doi:10.1186/s12934-021-01668-2)

Additional file 1 to: Overproducing the BAM complex improves secretion of difficult-to-secrete recombinant autotransporter chimeras

Trang H. Phan[1], Coen Kuijl[2], Dung T. Huynh[1], Wouter S. P. Jong[3], Joen Luirink[1,3], Peter van Ulsen[1]

1) Department of Molecular Microbiology, Amsterdam Institute of Molecular and Life Sciences, Vrije Universiteit Amsterdam, Amsterdam, The Netherlands
2) Medical Microbiology and Infection Control, Amsterdam Institute of Infection & Immunity, Amsterdam UMC, Amsterdam, The Netherlands
3) Abera Bioscience AB, Solna, Sweden

This file contains Figure S1 and S2.

Figure S1. Plot of the growth curves of BL21 DE3 expressing either HbpD-ESAT6, HbpD-ELL alone or together with BAM from pJH114. The graph shows that production of the HbpD chimera and the BAM complex influences cell growth. Data obtained from a 96-well format growth experiment. One representative experiment of three is shown.

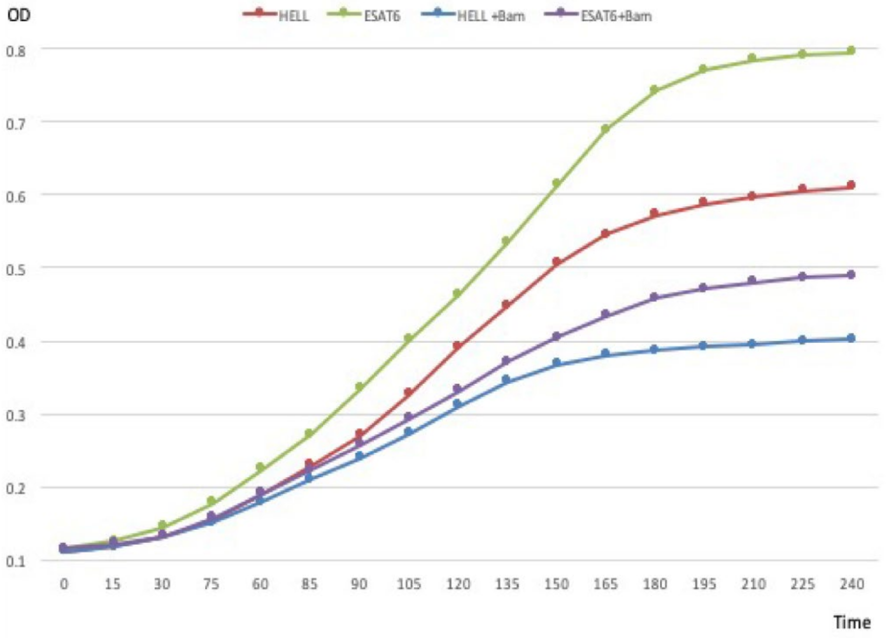

Figure S2. Fluorescence image of a semi-native SDS-PAGE gel. Cultures co-expressing HbpD-SpT2, HbpD-SpT2-LL or HbpD-SpT2-LL with BAM were incubated with SpC2-mScarlet or not to allow for coupling of the fluorescent protein to the HbpD-SpT2 variants exposed on the cell surface. Samples were then taken and dissolved in semi-native sample buffer and run on semi-native gels and imaged for fluorescence of mScarlet (Top). For reference, the gels were then stained with Coomassie Brilliant Blue and scanned (Bottom). The coupled Hbp-bands are indicated (<adduct), as well as the position of non-coupled SpC2-mScarlet (* SpC2-mScarlet). The intensity of mScarlet-labelled bands increases when HbpD-SpT2-LL was co-expressed with BAM. The whole cell suspensions used for this image are also used for Fig. 3B.

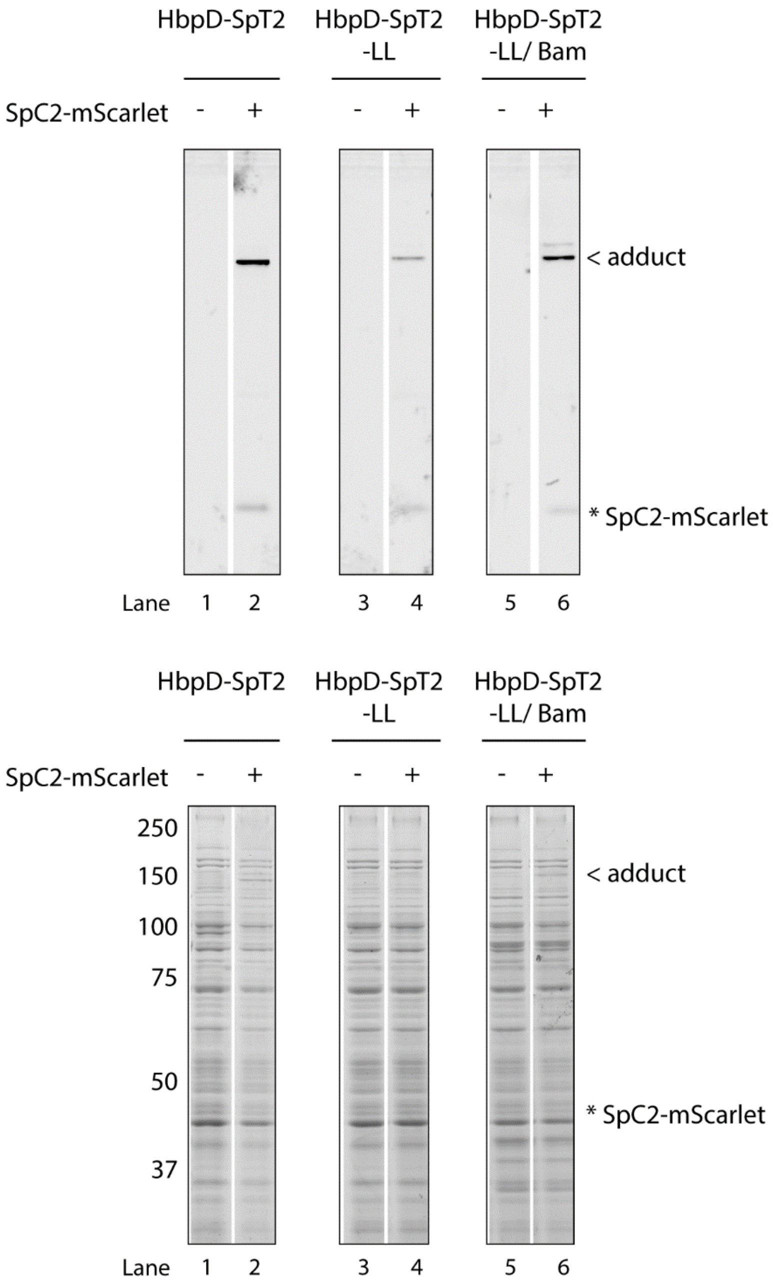

Supplement: Supplementary file 1 — Additional file 1: Figure S1. Plot of the growth curves of BL21 DE3 expressing either HbpD-ESAT6, HbpD-ELL alone or together with BAM from pJH114. Figure S2. Fluorescence image of a semi-native SDS-PAGE gel with samples of cultures co-expressing HbpD-SpT2, HbpD-SpT2-LL or HbpD-SpT2-LL with BAM that were incubated with SpC2-mScarlet or not to allow for coupling of the fluorescent protein to the HbpD-SpT2 variants exposed on the cell surface. [file 12934_2021_1668_MOESM1_ESM.pdf]
